# Transient Acquired Amegakaryocytic Thrombocytopenia in the Setting of Severe Sepsis: A Case Report

**DOI:** 10.7759/cureus.64072

**Published:** 2024-07-08

**Authors:** Steven B Barker, Aleksandra Ignatowicz, Andrew Strike, Christopher Chew, Jennifer K Anderson

**Affiliations:** 1 Internal Medicine, Northeast Georgia Medical Center Gainesville, Gainesville, USA; 2 Internal medicine, Northeast Georgia Medical Center Gainesville, Gainesville, USA; 3 Critical Care, Louisiana State University Health Sciences Center, Shreveport, USA; 4 Hematology and Oncology, Northeast Georgia Medical Center Gainesville, Gainesville, USA

**Keywords:** hematopathology, myeloid to erythroid ratio, severe sepsis, transient amegakaryocytosis, acquired amegakaryocytosis, thrombocytopenia

## Abstract

Acquired amegakaryocytic thrombocytopenia (AATP) is a rare disorder in which severely low platelet levels occur due to reduced or complete absence of megakaryocytes in the bone marrow. The pathophysiology of this disease is not fully understood, although anti-thyroid peroxidase antibodies (anti-TPO) binding to cellular-myeloproliferative leukemia (c-mpl) receptors is a proposed mechanism. Currently, no standard published guideline for treatment exists, but immunosuppressive therapies have been used based on the proposed mechanism and associated conditions. We present a case of a 57-year-old male who presented to the hospital with a 3-day history of progressive weakness and dysphagia. He had recently been discharged from an outside health system after evaluation for suspected gastrointestinal bleeding, although esophagogastroduodenoscopy and colonoscopy did not uncover a source of bleeding. Fifteen days later, he was admitted to our hospital for septic shock and acute renal failure with suspected lower gastrointestinal bleeding (melena on presentation). He was found to have a rapidly declining platelet count with a nadir of 0. Due to severe thrombocytopenia, filgrastim was administered. A bone marrow biopsy revealed findings consistent with amegakaryocytosis with otherwise preserved cell lines. Hematologic labs improved with the initiation of appropriate treatment for severe sepsis. After performing an extensive workup, the likely etiology of transient AATP in this case was severe sepsis-induced immune dysregulation and bone marrow suppression.

## Introduction

Amegakaryocytic thrombocytopenia (AAT) is a rare hematologic disorder presenting with fewer than 100 published cases. It is characterized as a severe thrombocytopenia with reduced or complete absence of megakaryocytes in the bone marrow and normal hematopoiesis [1]. The acquired variation, or acquired amegakaryocytic thrombocytopenia (AATP), is often seen later in life with previously documented associations in pregnancy, autoimmune disorders, viral infections including hepatitis C virus (HCV) and Epstein-Barr virus (EBV), environmental toxins such as benzenes, lymphoproliferative disorders and nutritional deficiencies including vitamin B12 [2]. Although the etiology remains unknown, several proposed AATP mechanisms include suppression of megakaryocyte maturation by exogenous or endogenous agents and early manifestation of stem cell abnormalities [3]. The most supported mechanisms include an immune-mediated process (endogenous) with antibody production specifically to platelet production regulator thrombopoietin (THPO) receptors. THPO is responsible for all stages of platelet production. THPO binds to the THPO receptor on megakaryocytes and hematopoietic stem cells, resulting in megakaryocytes’ proliferation, differentiation, and maturation into platelets. The presence of anti-THPO antibodies binding to the THPO receptor blocks THPO function. This theory has been supported by in vitro studies showing inhibition of megakaryocytic lineages like patients with AAT [4,5]. Patients with systemic lupus erythematosus (SLE), HCV, and systemic sclerosis have been found to have THPO receptor autoantibodies, resulting in the development of AATP. The condition progresses with complications of aplastic anemia, hemolytic anemia, and myelodysplastic syndrome without prompt treatments with aggressive immunosuppressive therapies [4-7].

Although there are several proposed algorithms for treating AATP, depending on etiology, there is no standard published guideline. Several recommended treatments include removal of the offending agent, antiviral therapy, or immunosuppression for patients with anti-megakaryocyte antibodies or T-cell mediated inhibition of megakaryopoiesis. Several studies have proposed using cyclosporine, anti-thymocyte globulin (ATG), rituximab, eltrombopag, intravenous immunoglobulin, steroids, or bone marrow transplant in refractory cases [5,8]. According to multiple case reports, cyclosporine as a monotherapy appears to be the most effective treatment course [3]. Prompt treatment is recommended due to the risk of complications and to prevent a relapsing-remitting disease state [3].

## Case presentation

The patient is a 57-year-old male with a history of hypertension, chronic kidney disease, suspected rheumatoid arthritis not on immunosuppressive therapy, and known sacral decubitus ulcer who presented to the emergency department due to progressive weakness and a 3-day history of dysarthria and dysphagia. Fifteen days prior, he was discharged from the hospital for anemia suspected to be due to a gastrointestinal bleed, although he did not have signs or symptoms of gastrointestinal bleeding at that time. During that hospitalization, his blood work revealed a hemoglobin of 6.6 g/dL, hematocrit of 21.5%, platelet 409 K/µL, and white blood cell count of 7.0 K/µL. Ferritin was 181.90 ng/mL, iron 13 µg/dL, total iron binding capacity 191 µg/dL, and iron saturation 7%. He received a blood transfusion of two units (700 mL) of packed red blood cells for a hemoglobin of less than 7.0 g/dL. During his first hospitalization, he underwent esophagogastroduodenoscopy and colonoscopy, which showed mild erosive esophagitis and tubulo-villous adenoma in the sigmoid colon, respectively. A polypectomy was performed during his colonoscopy.

Fifteen days later, he presented to our hospital with subsequent deterioration and was noted to be encephalopathic and found to have a melanotic stool on inspection. Vitals were notable for blood pressure 81/50 mmHg, heart rate 86 bpm, respiratory rate 32 bpm, oxygen saturation 87% on room air, and afebrile with a temperature of 97.3°F. Initial laboratory findings on presentation are found in (Table 1). He received two units of packed RBCs and was admitted to the ICU for further care. Continuous renal replacement therapy was initiated for acute renal failure and hyperkalemia. A CT of the brain was negative for signs of acute intracranial bleeding. A CT of the chest, abdomen, and pelvis was unremarkable as well. Ultimately, he was treated for severe sepsis.

**Table 1:** Lab values on the day of admission.

Lab	Lab Value	Reference Range
Hemoglobin	5.0 g/dL	14.0-18.0 g/dL
Hematocrit	15.3%	42.0-52.0%
WBCs	15.0 K/µL	4.8-10.8 K/µL
Platelets	522 K/µL	130-400 K/µL
BUN	217 mg/dL	6-20 mg/dL
Creatinine	5.52 mg/dL	0.6-1.3 mg/dL
Potassium	7.7 mEq/L	3.7-5.2 mEq/L
Lactic acid	12.30 mmol/L	0.5-2.2 mmol/L
CO_2_	<10 mEq/L	23-29 mEq/L
Anion gap	>19 mmol/L	4-12 mmol/L
Bilirubin	3.0 µmol/L	2-21 µmol/L
Alkaline phosphatase	138 U/L	30-120 U/L
Venous blood gas		
pH	7.189	7.32-7.42
pCO_2_	19.3 mmHg	41.0-51.00 mmHg
HCO_3_	7.1 mmol/L	24.0-28.0 mmol/L

**Figure 1:**
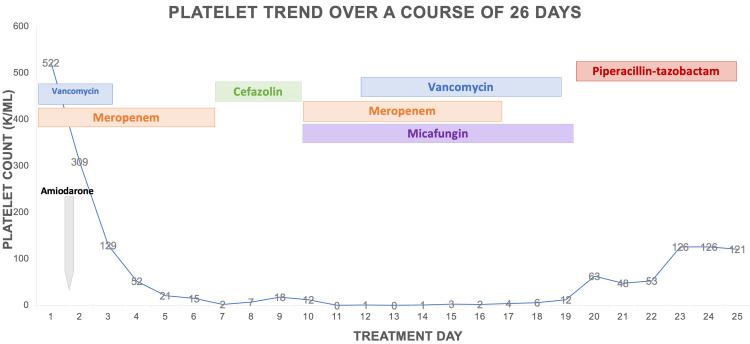
The platelet trend from admission showed a rapid decrease in platelet level from day 1 to a platelet level of 0 on day 7.

**Figure 2:**
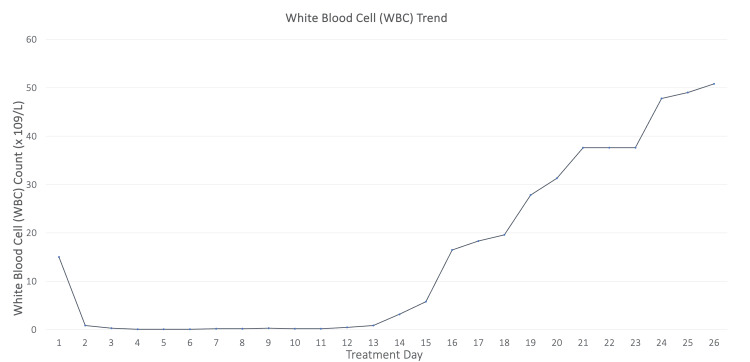
The WBC trend from admission over 26 days. From admission until day 11, a significant decrease in WBC levels was noted, followed by a gradual uptrend in levels.

On hospital day 2, he developed severe leukopenia and thrombocytopenia and was placed on an amiodarone drip for atrial fibrillation with rapid ventricular response after cardiac arrest. On hospital day 3, the patient was started on filgrastim due to worsening pancytopenia and severe neutropenia (Figures 1-2).

The patient’s shock improved with volume replacement; however, he started having increased vasopressor requirements. He was started on broad-spectrum antibiotics with vancomycin, meropenem, and micafungin, as no source of infection was identified. Initial blood cultures collected on admission were negative. Repeat blood cultures on day 3 were positive for multiple staphylococcus species, including methicillin-sensitive *Staphylococcus aureus* (MSSA). Antibiotics were adjusted to cefazolin at that time. As part of an effort to achieve infection source control, he was noted to have an enlarged and inflamed left olecranon bursa and left knee. Bursa arthrocentesis revealed monosodium urate crystals. He was started on allopurinol and prednisone for an acute gout flare. A left knee arthrocentesis was postponed due to platelet levels.

**Figure 3:**
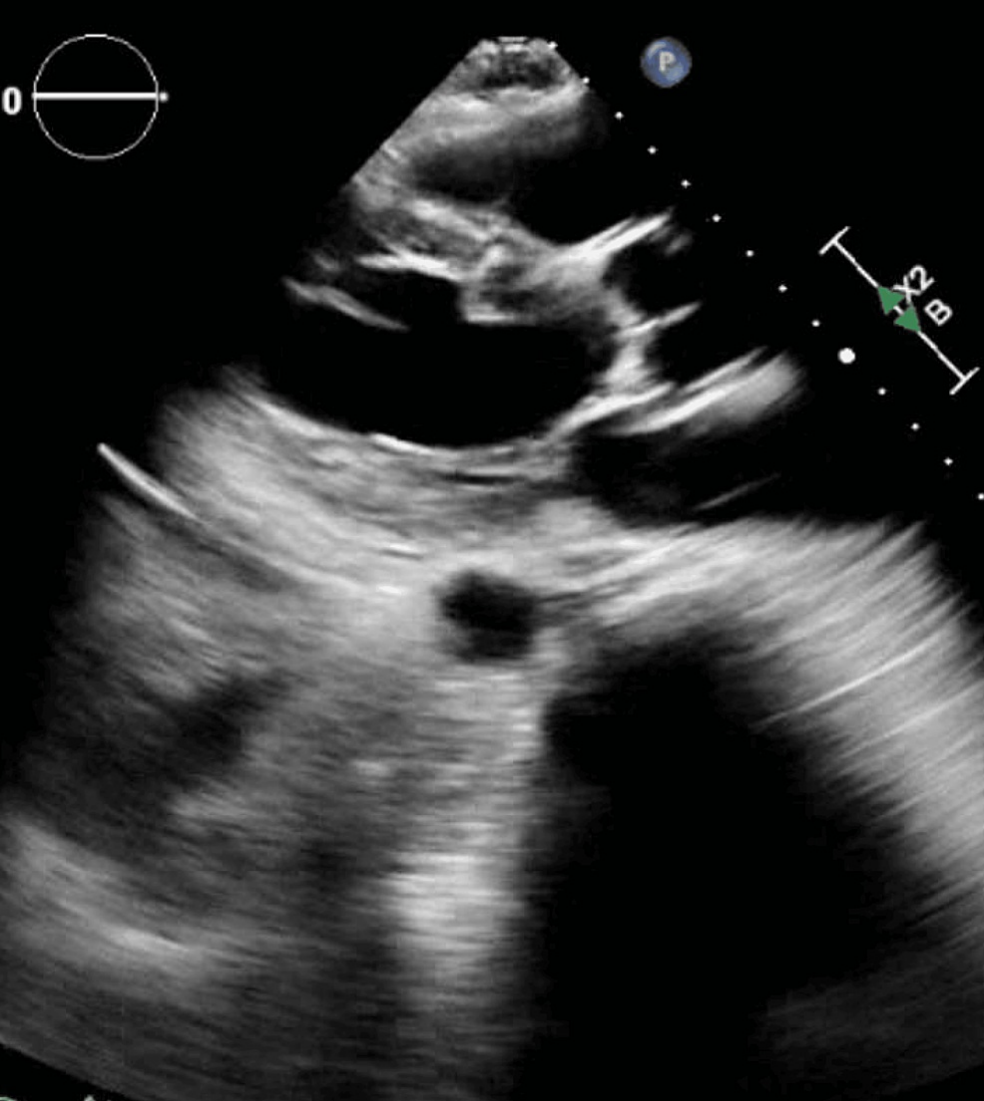
Transthoracic echocardiogram which did not show valvular vegetations.

Hematology-oncology was consulted for an evaluation of pancytopenia. At this point, the differential included pancytopenia secondary to medications, including antibiotics with vancomycin and meropenem and IV proton pump inhibitors. The patient had exposure to bone marrow suppressive medications, such as allopurinol and amiodarone, but not until the patient’s cell counts had already started to decline. A disseminated intravascular coagulation (DIC) panel was negative. Direct antiglobulin test (DAT) and lactate dehydrogenase (LDH) were normal. Folate and B12 levels at 1.1 and 202 units were supplemented accordingly. Testing for EBV, HIV, and viral hepatitis was negative. He had a transesophageal echocardiogram (Figure 3) which was negative for signs of valvular vegetations or valvular pathologies.

**Figure 4:**
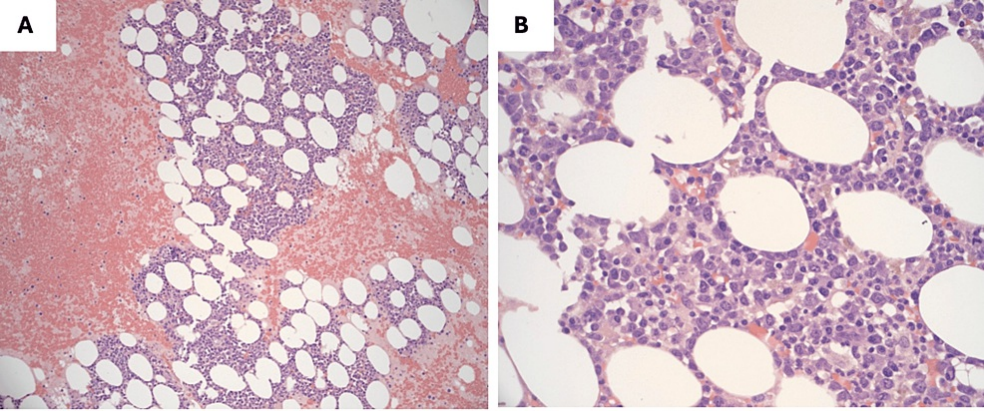
Bone marrow core biopsy at low power (100x) magnification (A) and high power (1000x) magnification (B) revealed hypercellular marrow with a myeloid-to-erythroid ratio of at least 5:1, a marked decrease in megakaryocytes (near absent) and approximately 30% polytypic plasma cells. No overt malignancy, blasts, or dysplasia was identified. The marked increase in the myeloid-to-erythroid ratio represented a reactive condition due to the patient’s critical medical condition and infections.

**Figure 5:**
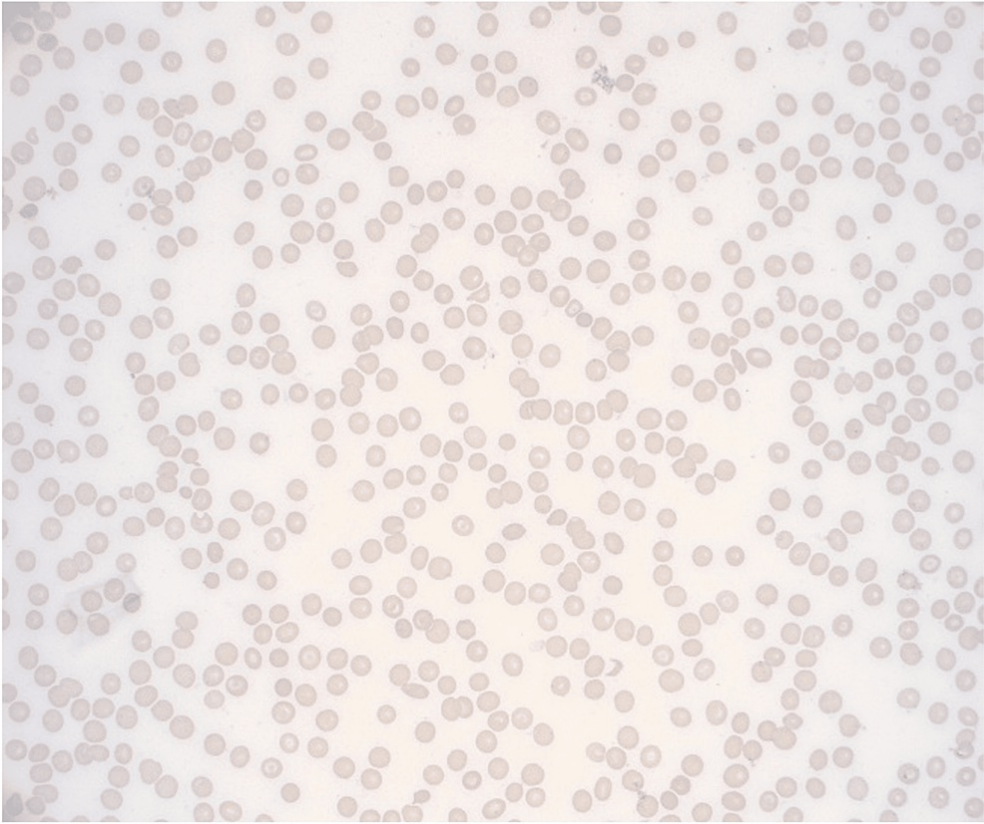
Peripheral blood smear with severe thrombocytopenia, microcytic, and hyperchromic anemia. No blasts were identified.

Flow cytometry and bone marrow biopsy were ordered to rule out occult malignancy or drug-induced aplastic anemia. The bone marrow was hypercellular with a myeloid-to-erythroid ratio of at least 5:1 (due to filgrastim administration) with near absent or absent megakaryocytes (Figures 4-5). Flow cytometry showed 30% polytypic plasma cells, but this finding was only seen on flow cytometry. Immunostains for CD138, CD34, CD15, and CD71 support the findings above, although no imaging was provided. No amyloid deposition is seen on Congo Red. A peripheral blood smear revealed severe thrombocytopenia and microcytic and hyperchromic anemia (Figure 4). The patient required multiple platelet and blood transfusions for worsening pancytopenia despite filgrastim injections.

**Figure 6:**
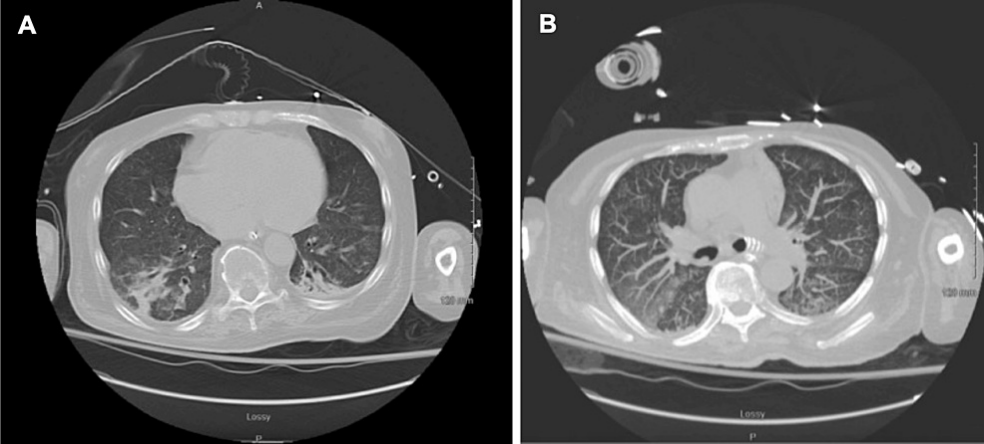
Tree-in-bud phenomenon seen on chest CT without contrast on day 25 of hospitalization.

The patient progressed through a lengthy hospital course complicated by septic shock requiring vasopressor support and mechanical ventilation due to respiratory failure. The patient was treated with vancomycin (day 1-3, 12-19, 32-33), meropenem (day 1-7, 10-19, 31), cefazolin (day 7-10), micafungin (day 10-19), piperacillin-tazobactam (day 19-31), gentamycin (day 19), tobramycin (day 28-30, 33), ceftazidime-avibactam (day 32-33), cefiderocol (day 34-46), ampicillin-sulbactam (day 40-55), ciprofloxacin (day 47-56), according to culture results. On day 10, the patient clinically deteriorated with worsening shock, requiring aggressive vasopressor support. Repeat blood cultures were positive for bacillus species and pan-sensitive *Pseudomonas aeruginosa*. Meropenem was restarted on day 10 of hospitalization. Despite appropriate treatment, blood cultures on days 17, 21, and 23 continued to grow *P. aeruginosa*. Repeat CT imaging of the chest (Figures 6A-6B) demonstrated diffuse tree-in-bud nodularity throughout both lungs and a cavitary lesion in the left upper lobe.

Respiratory cultures resulted in heavy *P. aeruginosa* growth. The source of infection at the time was most likely due to Pseudomonas pneumonia or a sacral decubitus infection. Antibiotics were later adjusted to ceftazidime-avibactam on day 32, given the growth of multidrug-resistant Pseudomonas and *Enterococcus faecalis* from the sacral decubitus ulcer and respiratory cultures. The antibiotic regimen was changed again to cefiderocol due to a lack of clinical improvement. The patient significantly improved with the initiation of cefiderocol on day 34, and his oxygen requirements were gradually weaned. He was treated with a total of 13 days of cefiderocol before transitioning to ciprofloxacin and ampicillin-sulbactam at discharge to a long-term acute care facility.

## Discussion

AATP is a rare condition described in less than 100 case reports. There are several proposed mechanisms ranging from direct megakaryocyte destruction via drug exposure and anti-THPO antibodies. Still, the differential also includes autoimmune etiologies such as rheumatoid arthritis and SLE, infectious etiologies, or idiopathic causes. Diagnosis of AATP usually consists of addressing the suspected underlying etiology, whether infectious, immune-related, or drug-related. As there are no standardized treatment guidelines, treatment should be prompt and focused on using THPO receptor agonists, immunosuppressives, or, in our case, treatment of severe sepsis [9-12].

The patient underwent an extensive cytopenia workup for multiple etiologies, and AATP was the diagnosis of exclusion once the bone marrow results were available. He was found to have vitamin B9 and B12 deficiencies and was supplemented accordingly without improvement of the cytopenia. Second, rheumatoid arthritis should be excluded before making a diagnosis of AATP. Before hospitalization, the patient was suspected to have rheumatoid arthritis due to joint deformities resulting in significant debility, although no formal workup was done before his hospitalization. The joint aspiration was diagnostic of gout but without any findings suggesting rheumatoid arthritis. Peripheral blood smear showed a near absence of platelets without enlarged forms, anisopoikilocytosis consistent with transfusion, 0-1 schistocytes per high-powered field, and enlarged hypersegmented neutrophils with toxic granulation but no other immature or abnormal leukocyte forms. Testing for consumptive coagulopathies such as DIC, heparin-induced thrombocytopenia (HIT), hemolytic uremic syndrome (HUS), and thrombotic thrombocytopenic purpura (TTP) was negative. There was no evidence of enlarged platelets on peripheral blood smear. Evan’s syndrome, immune thrombocytopenia (ITP), and autoimmune hemolytic anemia were unlikely as splenomegaly, immune destruction of red blood cells, and lymphadenopathy were absent. The patient had no history of chemotherapy or radiation treatment [13]. The patient was not taking supplements or immunosuppressives, which could have contributed to his pancytopenia. Immune suppression during the hospitalization was also not pursued due to septic shock. The bone marrow biopsy results showed changes suggestive of megakaryocytic destruction in the setting of the patient’s critical illness as the etiology of his thrombocytopenia. Specific antibody testing, such as anti-THPO receptor antibodies or THPO levels, was not performed in this case, but this did not exclude the diagnosis as this is only one of several proposed pathophysiological mechanisms of AATP. Although this testing was not performed, the clinical presentation, absence of other causes of thrombocytopenia, and near absent megakaryocytes on bone marrow biopsy point toward a diagnosis of AATP. The etiology of AATP in this case was most likely due to severe sepsis rather than cytotoxic medication use such as amiodarone, antibiotics, allopurinol, or an autoimmune process. The pancytopenia improved and ultimately resolved with the treatment of severe and multiple infections with the appropriate antibiotics, given significant drug resistance.

## Conclusions

Sepsis is known to cause new onset thrombocytopenia from decreased platelet production from sepsis-induced immune dysregulation or other factors such as consumption, sequestration, or immune-mediated platelet destruction. Sepsis can induce platelet activation or functional defect that results in DIC, secondary thrombotic microangiopathy, or TTP. However, there are no reports of sepsis causing AATP. Our case is considered a rare presentation of transient AATP secondary to severe sepsis.
